# Percutaneous device closure of iatrogenic right ventricular perforation during attempted ventricular septal defect closure: a case report

**DOI:** 10.1093/omcr/omaf287

**Published:** 2026-01-25

**Authors:** Muhammad Raza Sarfraz, Saqlain Anwar, Nadeem Sadiq, Yumna Shariff, Ahmad Hassan, Hassan Waqar, Zohab Ahmed, Kamil Ahmad Kamil

**Affiliations:** Department of Medicine and Surgery, Allied Hospital, Faisalabad Medical University, Sargodha Road, Faisalabad 38000, Punjab, Pakistan; Department of Medicine, PNS Shifa Hospital, Main Korangi Road, Clifton, Karachi 75600, Sindh, Pakistan; Department of Pediatric Cardiology, PNS Shifa Hospital, Main Korangi Road, Clifton, Karachi 75600, Sindh, Pakistan; Department of Pediatrics, Aga Khan University Hospital, Stadium Road, P.O. Box 3500, Karachi 74800, Sindh, Pakistan; Department of Medicine, Aga Khan University Hospital, Stadium Road, P.O. Box 3500, Karachi 74800, Sindh, Pakistan; Department of Medicine, PNS Shifa Hospital, Main Korangi Road, Clifton, Karachi 75600, Sindh, Pakistan; Department of Medicine, Aga Khan University Hospital, Stadium Road, P.O. Box 3500, Karachi 74800, Sindh, Pakistan; Medical Faculty, Malalay Institute of Higher Education, 2nd Aino Mina, East, 28th Sub-Street, Kandahar 3802, Afghanistan

**Keywords:** cardiology and cardiovascular systems, emergency medicine, paediatrics

## Abstract

Cardiac perforation is a rare but life-threatening complication of transcatheter cardiac procedures, particularly in children where tissue fragility increases risk. We present a rare case of iatrogenic right ventricular perforation during transcatheter ventricular septal defect closure in a pediatric patient. During the procedure, inadvertent withdrawal of the Terumo guidewire caused perforation of the right ventricular apex, leading to cardiac tamponade and cardiopulmonary arrest. Emergency pericardiocentesis with pigtail catheter placement and autotransfusion restored hemodynamic stability, after which transcatheter repair was selected over surgical intervention due to the patient’s critical condition. An 8 mm Shanghai Shape Memory Alloy occluder was successfully deployed to seal the perforation, resulting in complete hemostasis and a stable recovery. This case highlights that transcatheter device closure can serve as a life-saving alternative to emergent surgery for iatrogenic cardiac perforations in children, offering a minimally invasive solution in high-risk scenarios.

## Introduction

Percutaneous device closure is a minimally invasive option for ventricular septal defects (VSD), with faster recovery and lower morbidity than surgery but carrying rare, potentially fatal risks such as myocardial perforation [1, 2]. The aim of this report is to describe a pediatric case of iatrogenic right ventricular perforation (RVP) during VSD closure, successfully managed with transcatheter repair, highlighting percutaneous closure as a potential life-saving alternative to emergency surgery.

## Case report

**Figure 1:**
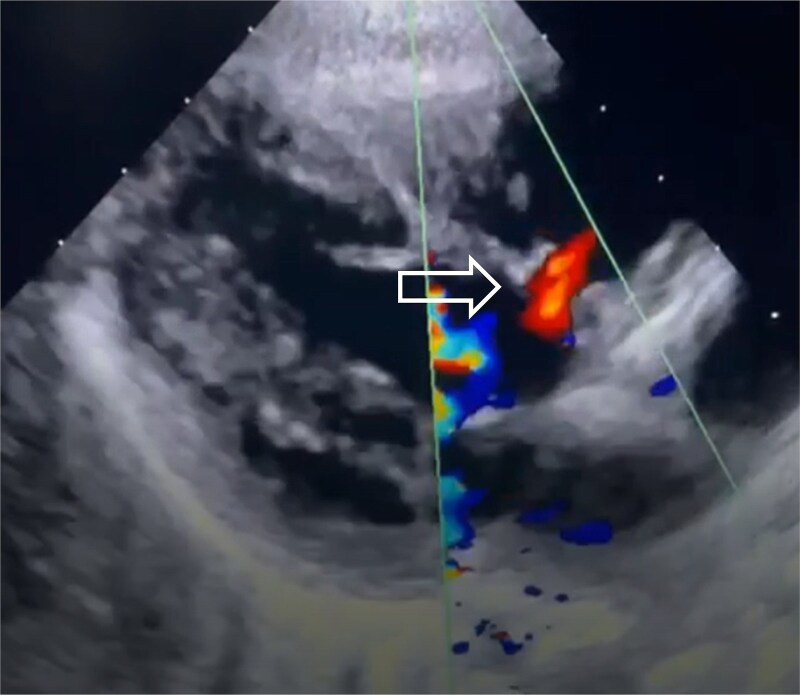
Baseline echocardiographic image demonstrating a moderate outlet muscular ventricular septal defect with a diameter of 4.5 mm.

A 3-year-6-month-old girl from rural Sindh, Pakistan, weighing 12 kg, with height 95 cm and head circumference 49 cm, presented to our pediatric outpatient department with recurrent cough and respiratory infections since infancy. Parents denied fever, edema, chest pain, seizures, or squatting episodes; family history was negative for cardiac disease. Developmental assessment showed delays in gross motor, speech, and personal-social milestones, likely due to the congenital condition and prolonged illness. On examination, the child appeared well without cyanosis, conjunctival congestion, chest retractions, or nasal flaring. Vital signs were normal with no clinical evidence of pulmonary hypertension. Respiratory examination revealed bilateral vesicular breath sounds without added sounds. Cardiac examination demonstrated a grade III pansystolic murmur at the lower left sternal border. Abdominal and neurological findings were unremarkable. Peripheral oxygen saturation ranged from 95–98%. Laboratory results showed hemoglobin 10 g/dl, hematocrit 32%, and platelet count 270 000/μl. Renal and hepatic function tests were normal. Chest radiography demonstrated cardiomegaly with increased pulmonary vascular markings. Echocardiography confirmed a moderate outlet muscular VSD measuring 4.5 mm with left-sided volume overload (Fig. 1).

**Figure 2:**
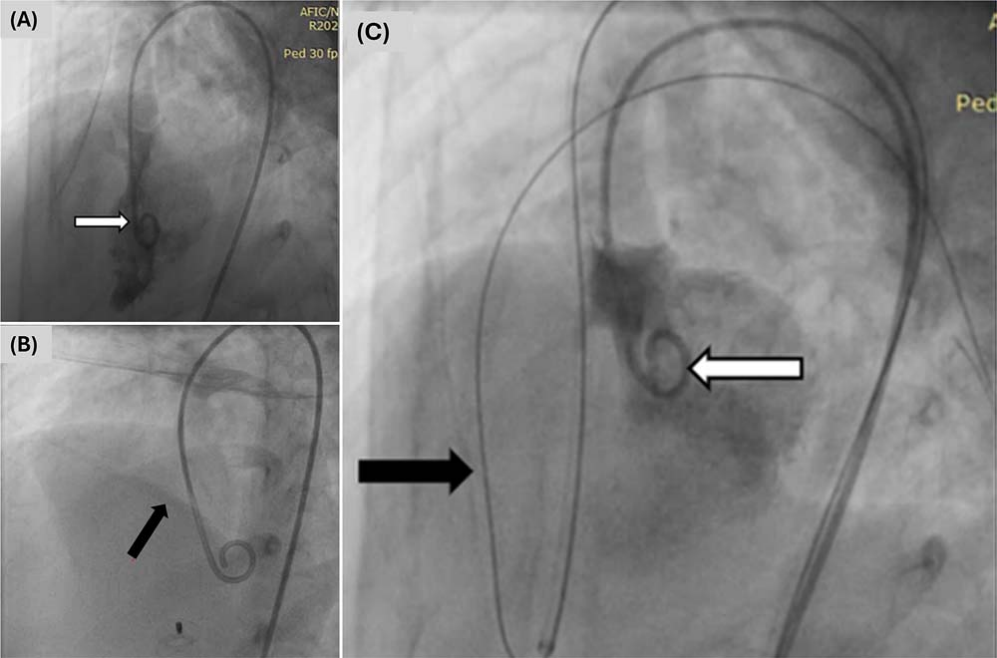
Angiographic images showing (A) ventricular septal defect, (B) pulmonary-to-systemic flow ratio of 2.2:1 and pulmonary vascular resistance of 2.5 wood units/m^2^ and (C) Terumo guidewire (black arrow) with long sheath delivery system positioned in the right ventricle (white arrow).

Based on these findings, the patient was scheduled for elective percutaneous VSD repair under general anesthesia. Unfractionated heparin (100 U/kg) was administered with activated clotting time monitoring. Left ventricular angiography confirmed a moderate outlet muscular VSD with a pulmonary-to-systemic flow ratio of 2.2:1 and pulmonary vascular resistance of 2.5 Wood units/m^2^. The defect was crossed using a 0.35-inch Terumo (Radifocus™) 260 cm guidewire with a Judkins Right 5F catheter, and the wire was positioned in the left pulmonary artery. A retrograde approach was chosen for device closure with a Multifunctional Occluder (Lifetech™ Konar-MF). A 6F long sheath was then advanced over the guidewire into the right ventricle (Fig. 2).

**Figure 3:**
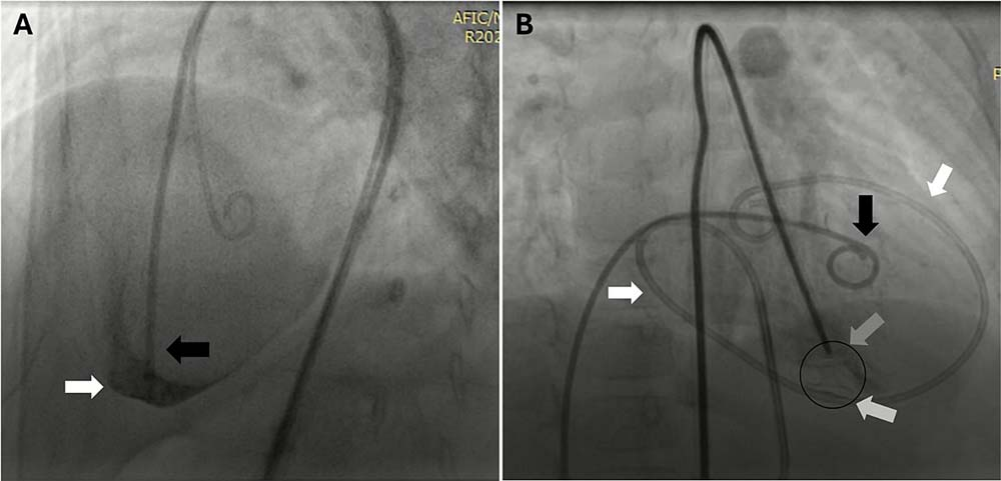
Fluoroscopic images showing right ventricular perforation and device deployment. (A) Right ventricular perforation (black arrow) with the long sheath delivery system extending into the pericardium (white arrow). (B) First pigtail catheter positioned in the right ventricle (black arrow) and second pigtail catheter in the pericardium (white arrow), with the proximal end of the device attached to the delivery system (dark grey arrow) and the distal end already deployed (light grey arrow).

During withdrawal of the Terumo guidewire, the delivery system inadvertently perforated the RV apex (Fig. 3A). The patient rapidly developed hemodynamic instability, first showing pulsus paradoxus on arterial tracing, followed by cardiovascular collapse. Transthoracic echocardiography (TEE) revealed a large pericardial effusion with tamponade, and contrast confirmed sheath entry into the pericardial space. The patient progressed to cardiopulmonary arrest, requiring immediate advanced cardiac life support. Emergent pericardiocentesis was performed, and a pigtail catheter was left for continuous drainage. A second pigtail catheter was placed into the RV, allowing complete evacuation of the hemopericardium (Fig. 3B). Concurrent autotransfusion of aspirated pericardial blood was initiated to maintain hemodynamic stability. After multidisciplinary consultation, surgical repair was deemed prohibitively risky given the patient’s tenuous condition, so transcatheter occlusion of the iatrogenic defect was pursued under echocardiographic and fluoroscopic guidance. The initial 6F sheath was exchanged for an 8F long sheath to allow device delivery. An 8-mm Shanghai Shape Memory Alloy (SHSMA) muscular VSD occluder (double-disc design, LEPU Medical Technology, Beijing, China) was advanced and deployed, with the distal disc positioned in the pericardial space and the proximal disc in the right ventricular cavity, effectively sealing the perforation. Right ventriculography confirmed optimal device placement at the apex with complete occlusion and no contrast extravasation (Fig. 3B). Following confirmation of hemostasis, the device was released from the delivery cable. Pericardial drainage catheters were left in place prophylactically against recurrent bleeding. The patient was transferred to the coronary care unit for intensive monitoring, and post-procedural TTE confirmed no reaccumulation of pericardial fluid.

**Figure 4:**
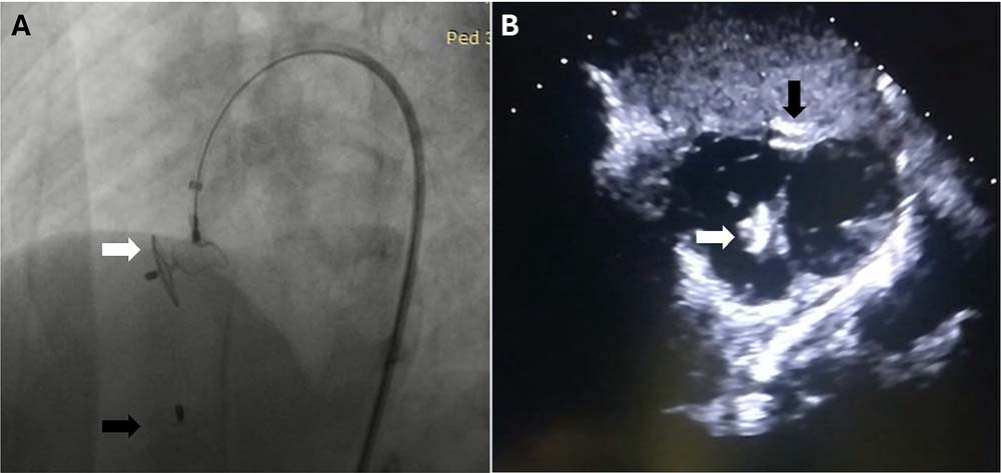
Post-procedural imaging demonstrates successful device placement. (A) Angiogram showing two well-positioned devices at the right ventricular apex (black arrow) and ventricular septum (white arrow). (B) Two-dimensional echocardiographic image confirming optimal device positioning at the right ventricular apex (black arrow) and ventricular septum (white arrow) with no residual leakage.

Post-procedure TEE showed no pericardial fluid reaccumulation. The pericardial drains were removed 24 h later after confirming absence of reaccumulation. Serial echocardiography at 24 and 48 h revealed no residual effusion, and the patient was discharged on day three. At eight weeks, follow-up echocardiography confirmed absence of effusion. Three months later, elective VSD closure was successfully performed using a 10/8 mm MFO (Lifetech™ Konar-MF) device under echocardiographic and fluoroscopic guidance with an arteriovenous loop (Fig. 4). The patient was discharged the next day, and subsequent imaging confirmed stable device positions without complications.

## Discussion

We report a case of RVP during percutaneous VSD closure in a 3-year-6-month-old girl, successfully managed with transcatheter occlusion using a muscular VSD occluder. The complication resulted from inadvertent sheath advancement beyond the guidewire tip during withdrawal, causing cardiac tamponade and hemodynamic collapse. RVP during percutaneous VSD closure is rare but potentially fatal, particularly in children with small cardiac anatomy and thin ventricular walls [2, 3].

Although the diagnosis was evident from procedural context and immediate imaging, the differential diagnosis includes perforation of the right atrium, left ventricle, or great vessels [4]. Contrast extravasation at the RV apex confirmed the diagnosis of iatrogenic RV apical perforation. In this case, transcatheter occlusion was chosen over surgery due to prohibitive risk, with the distal disc deployed in the pericardial space to achieve hemostasis. Due to its rare documentation in literature, this approach shows that percutaneous closure can be a viable alternative to surgery in unstable cases, with staged definitive repair highlighting the value of individualized, multidisciplinary management in complex pediatric interventions.

Conventional management of such perforations has relied on emergency surgery, typically using purse-string sutures or patch closure [5]. Few cases are reported, as small vessel access limits standard cannulation and the immature myocardium poorly tolerates prolonged clamping or manipulation [6]. Although surgical repair remains definitive, it carries significant morbidity in this cohort, including arrhythmias, pleural complications, and prolonged ICU stays [7]. Recent literature shows a paradigm shift toward percutaneous closure of iatrogenic cardiac perforations. Temel MT et al. reported successful closure of an iatrogenic VSD in a 1-year-old using an Amplatzer Piccolo Occluder, avoiding sternotomy and highlighting minimally invasive options [8]. Studies demonstrate > 95% procedural success, minimal complications, and excellent three-year outcomes. In our case, no complications such as AV block or new atrial arrhythmias occurred, unlike a surgical series where 17% developed worsening aortic regurgitation [9]. The SHSMA occluder, with its flexible nitinol double-disc design, enables precise sealing while minimizing trauma to fragile pediatric myocardium.

This case highlights a novel approach to managing catastrophic complications in pediatric interventional cardiology. Successful transcatheter closure of RVP using a muscular VSD occluder, with the distal disc deployed in the pericardial space, is rarely reported in children. It demonstrates that percutaneous repair can be a life-saving alternative to high-risk surgery in hemodynamically unstable pediatric patients and should be considered as a minimally invasive option.
